# New products

**DOI:** 10.1155/S1463924690000050

**Published:** 1990


Journal of Automatic Chemistry Vol. 12, No. 1 (January–February 1990), pp. 41–47

### New products

### Thermal conductivity gas Analyser

The Caldos 5, a novel thermal conductivity gas analyser, is now available from Applied Automation, a member of the Hartmann & Braun Group. It can continuously and quantitatively determine the concentration of a single gas component in a binary or quasi-binary gas mixture.

The Caldos 5 gas analyser can measure a wide variety of inert or highly corrosive gases. Commonly measured components include Ar in $O_2$, $H_2$ in Ar, $H_2$ in $N_2$, $H_2$ in blast furnace gas, $CO_2$ in $N_2$ or air, $SO_2$ in $N_2$ or air, $NH_3$ in $H_2$ or air, $H_2$ in $Cl_2$, $H_2$ in HCl, $Cl_2$ in HCl, HCl in $Cl_2$, and $CH_3$ in $CO_2$.

Applications for the Caldos 5 include gas purity monitoring, continuous monitoring of flue and inert gases, and blast furnace and converter exhaust gas monitoring. The analyser can also be used for process monitoring in air separation and chlor-alkali electrolysis plants, as well as in ammonia synthesizing plants and for room air monitoring.

The Cados 5's output signal is proportional to the volume per cent of the gas being analysed so it can also be used as a control signal generator for limiting gas concentrations. Sample gas flows through two opposite chambers, each of which contains a temperature-dependent resistor of a bridge circuit. The two other resistors of the bridge circuit are in contact with a zeroing reference gas. When resistors are heated, slight differences in thermal conductivity of sample and reference gases disrupt the temperature-dependent bridge balance. The resulting bridge diagonal voltage is then processed digitally to yield concentration.

The response time of the thermal conductivity gas analyser can be as low as 6 s. The Caldos 5 features rugged construction, long-life glass-encapsulated filament sensors, and automatic or manual range calibration. It can handle up to three internally selectable linear measuring ranges.

*Details from: Applied Automation/ Hartmann & Braun, PO Box 9999, Bartlesville, Oklahoma 74005-9999, USA. Tel.: 918 662 700.*

### Computer-controlled power supply

The MultiDrive XL is a versatile, easy-to-program power supply with unparalleled safety features.

MultiDrive XL is ideal for most electrophoretic techniques, including isoelectric focusing (IEF), SDS-PAGE, DNA sequencing, blotting (both tank and semi-dry techniques), and pulsed field gel electrophoresis. It supplies constant current to 400 mA, constant voltage to 3500 V, and constant power to 200 W. Nine methods, each containing nine steps, can be programmed and stored in the non-volatile memory.

MultiDrive XL offers a choice of four control modes: time, volt-hour integration, milliampere-hour integration, or the derivative of current with respect to time. Voltage-ramping can be programmed for optimal sample run-in. Four high voltage outlets allow four separations to run simultaneously.

Unique high voltage connectors with coded sleeves (patent pending), protect separation units against voltage overload and ensure personal safety. Other safety features include automatic power shut-off when ground leakage greater than 0·5 mA is detected, when an outlet plug is removed or if the thermostatic circulator fails.

*More information from: Pharmacia LKB Biotechnology AB, Electrophoresis Division, Box 305, S 161 26 Bromma, Sweden. Tel.: 46 8799 80 00.*

### New IRD

Hewlett-Packard's recent infrared detector (IRD), the HP 5865A, has a sensitivity believed to be several times greater than any other vapour-phase system currently available. Coupled with the recently-launched HP 5890 Series II GC and HP ChemStation controller, the new IRD – the successor to the original HP 5965A IRD – makes low-cost capillary GC/FTIR analysis both simpler and quicker to run.

The new IRD features an array processor that displays the total-response chromatogram and absorbance spectrum of each peak, and up to four selected wavelength chromatograms, in real time. This allows the chemist to monitor a run continuously for key compounds, ensuring optimum separation, characterization and identification.

The array processor increases the number of scans per minute, so enhancing measurement sensitivity – the IRD detects 5 ng of isobutyl methacrylate or dodecane at a signal-to-noise ratio of 30:1. Absorbance data can be stored instead of interferograms, requiring less disk storage space.

For fast data reduction, the HP 9000 Model 340 computer – designed specifically for instrument control and scientific data handling – has 4 Mbytes of core memory as standard. A large, high-resolution colour display makes it easier to view and move data on screen.

The enhanced HP ChemStation Revision 3.2 software offers many new capabilities, and provides a full set of GC/IRD applications for data acquisition, editing and reporting. Screen prompts assist in method building, and peak qualification procedures automatically confirm or deny compound identification. Expanded calculation routines allow automatic integration of chromatograms; and a variety of mathematical and algebraic functions are included.

The new, soft-key-driven graphics environment enhances the presentation of data. Peaks may be labelled, axes drawn, grids displayed in several formats and colour data specified. Notation can be added in a choice of character, font and size.

Soft-keys provide single push-button control of most operations, such as the choice of short, long or extended report formats. Custom report formats are created using the macro-writing facility. The new software includes file management routines to back up data files on disk or tape automatically.

*Enquiries to: Verena Haller, Hewlett-Packard SA, 150 Route du Nant-d'Avril, CH 1217 Meyrin (GE) 2, Switzerland.*

### Modular, expandable process control system

Contronic P, an application-proved distributed process control system with an established international base of users worldwide, is available in the US from Applied Automation, a member of the Hartmann & Braun Group.

Using standardized, modular components, Contronic P manages all process control functions, including continuous and discreet control. It can be structured to meet specific needs of a wide variety of industrial types and sizes.

Although sophisticated, the system was designed for use by control systems technicians without special computer experience. A layered menu structure makes system configuration and parameter input simple.

The Contronic P includes autonomous process control stations containing modularly extendable process interfaces linked to transmitters, signal transducers and process-correcting equipment. In addition, blocks for state correction, quantity acquisition, signal sequence acquisition, disturbance variable feedback, sequence control and dosing circuits are also available.

A serial bus system handles all communications data up to 127 stations at the rate of 1 MBaud. At the central operator station, equipped with multiple colour video display units, keyboards and light pens are used to select process points, to adjust set points, output or alarm variables, and to initiate logs and acknowledge messages.

The Contronic P also includes a computer interface station, coupling to higher-level mainframe systems for process optimization, planning and balancing.

*Details from: Applied Automation/Hartmann & Braun, PO Box 9999, Bartlesville, Oklahoma 74005-9999, USA. Tel.: 98 662 7000.*

### High-performance photometers

A new generation of non-dispersive infrared (NDIR) industrial photometers – the Uras 3 series – is available from Applied Automation. The instruments are intended for continuous measuring of a variety of infrared-absorbing gases, including acetylene, ammonia, carbon dioxide, carbon monoxide, carbon disulphide, ethylene, hydrogen cyanide, methane, nitric oxide, nitrous oxide and sulphur dioxide.

To ensure the Uras 3 provides accurate readings even in high-vibration environments, a lock-in technique (phase-selective rectification) is employed for electronic signal processing. Additional protection in severe operating conditions comes from the rugged cast light alloy, two-compartment case that is dust- and splash-proof.

Most adjustments can be made in place with no special tools. Should it be necessary, the entire optical system can be removed by simply loosening two socket-head screws, connectors and gas lines.

All models of the Uras 3 series are supplied with corrosion-resistant measuring cells, one linearized measuring range, an electrically isolated signal output of 0/4 to 20 mA or 0/2 to 10 V, and a power supply requirement of 110 V/60 Hz.

The measurement ranges and resolution vary with the specific type of gas being analysed. As an example, CO measuring ranges are 0–100 ppm with a reproducibility of 0·5 ppm.

*Details from Applied Automation (as above).*

### Intelligent sample changer

The SC-150 is an intelligent peripheral device which is compatible with the company's line of sequential ICP emission spectrometers, as well as its range of atomic absorption spectrometers (AAS). For AAS analyses, the ISC-150 automates the analysis of trace and ultratrace metals since it is compatible with both flame and furnace atomizers. The ISC-150 accommodates up to 150 samples plus an auxiliary rack for blank controls, standard preparation, and matrix modification. These preparatory functions can be executed by interfacing the ISC-150 with the company's PS-150 Prep Station. A flow-through rinse station is also available.

The ISC-150 is also available as an OEM product which can be configured as desired through microprocessor control.

*For more information contact Thermo Jarrell Ash Corp., 8E Forge Parkway, Franklin, Massachusetts 02038, USA. Tel.: 508 520-1880.*

### Computer-controlled workstation

The MilliLab 1A Workstation is controlled by an optional, external computer. The MilliLab 1A package is based on the latest generation of personal computers – the 386. It operates with a very clear, menu-driven format that can control up to four MilliLab Workstations and accommodates multiple terminals. As a true multi-tasking system, operators may edit or compile one method while running another. Samples may be processed serially, batch and automatically in the 'Solvent Saver' mode for minimum solvent consumption. A set of samples can be subdivided to run more than one method per group.

The MilliLab 1A contains a modem for on-line support and connection into a MilliLab Users' Bulletin Board. There is method and system validation, as well as error checking. User access control is also provided.

The MilliLab 1A is based on an NEC APC 601C 386 computer with 1·2 MB floppy and 40 MB hard disk, APC 650 1MB additional memory, one serial and one parallel port, keyboard, 1200 Baud Modem, the Xenix Operating System V for 386, MilliLab 1A Application Program for single workstation control, black and white or colour EGA monitor. Options available include a 2400 baund modem, DOS emulator, multiple system control, printer and extended-length cable for remote control.

The MilliLab 1A will be suitable for installations in which there will be substantial new methods work, multiple workstation operation or the requirement for hard copy documentation of methods and results.

A menu-driven format enables the user to work directly from the computer screen and keyboard without any knowledge of computer programming. It is self-prompting. Each choice automatically moves to the next level of the program and indicates the information which is required. All information is expressed in common laboratory and chemistry terminology. The user begins in the main menu, choosing from six categories:

Run method: Run existing methods which have been stored in the MilliLab 1A.

Edit method: Revise existing methods, or create additional methods.

Step method: Perform each step of a method as it is being reviewed or created to verify that the intended operations are being done. User initiates each step.

System maintenance: Enter or change instrument configuration.

Reports: Display, print and save methods and results.

Other: Access MilliLab User Bulletin Board, third-party programs for word processing, spreadsheet etc. and DOS emulator.

Adding computer control to the MilliLab Workstation has made automated sample preparation easier and more efficient. Multi-tasking capability with the MilliLab 1A configuration makes the MilliLab Workstation operate even more efficiently since some operations are performed simultaneously. Methods operated without computer control have been reduced by 25% to 40% in operating time.

The ability to batch samples or batch portions of a sample preparation operation, such as conditioning all solid phase extraction cartridges with one solvent before switching to the next, can significantly reduce operating time and solvent consumption. A comparison of solvent consumption and waste generation in a procedure for separating food colourings shows significant reduction in solvent usage with partial batching or the automated 'Solvent Saver' modes, based on processing 10 samples.

*Solvent usage (ml); dye separation method – 10 samples. 1A operating mode.*

| | Serial | Batch | Solvent saver |
|---|---|---|---|
| 20% MeOH | 134 | 89 | 80 |
| 100% MeOH | 278 | 188 | 179 |
| 100% $H_2O$ | 399 | 311 | 253 |
| Total waste | 527 | 301 | 226 |
| Total solvent | 811 | 588 | 512 |

Prior to running a method, the user can choose to have the MilliLab 1A do a system and method check, to ensure that the user has prepared the system with adequate cartridges, filters, tube sizes, solvents and reagents. As a program is running it is also possible to have an active runtime status report presented on the monitor screen.

All of the capabilities of the MilliLab Workstation for performing automated sample preparation are retained by the MilliLab 1A configuration. These include: filtration, solid phase extraction, liquid/liquid extraction, serial or single dilution, addition of standards and reagents, mixing, evaporation, trace enrichment, feeding analytical instruments or autosampler carousels.

*Details from Dave Collis, Waters Chromatography Division, Millipore (UK) Ltd, The Boulevard, Ascot Road, Croxley Green, Watford WD1 8YW, UK. Tel.: 0923 816375; fax: 0923 818297.*

### SmartCell

The SmartCell product line combines hardware and software to allow discrete units of work to be interfaced into a totally automated workstation. Varying degrees of laboratory automation are possible for example: limited instrument interfacing; specific application workstations; multiple workstation networks; and laboratory robotics.

*For more information contact Art Martin, Source for Automation, Commerce Park N3, 115 Cedar Street, Milford, Massachusetts 01757, USA.*

### Continuous monitoring of mercury in air

Working in association with the University of Ghent, Belgium, PS Analytical has developed a fully automated continuous monitoring analysis system for mercury in air. The Merlin Fluorescence Detector provides a very sensitive detection system, which, when linked with the PSA 10.511 Galahad Adsorber/Desorber system, allows rapid responses to changes in mercury level, even when low mercury concentrations are involved. Air is drawn over the specially designed trapping unit and the mercury collected as an amalgam. On completion of the required collection time, determined by the flow rate of air over the bed, valve manifold switches the gas flows so that the air contained in the system is flushed out with argon prior to the mercury being revaporized and fed to the Merling Detector for quantification.

PS Analytical has integrated the air monitoring system into its TouchStone software operating the adsorber/desorber and collecting the data automatically. An efficient means of calibrating the system in an absolute manner is also included in the method held on computer file. Detection of mercury down to the level of 10 pg per cubic metre is achieved. With such an approach, changes in mercury levels can be picked up with extremely short sampling times, i.e. in the order of 10–15 min. Mercury can also be measured in a range of other gaseous environments using similar technology.

*Further details from PS Analytical Ltd, Arthur House, B4 Chaucer Business Park, Watery Lane, Kemsing, Sevenoaks, Kent TN15 6QY, UK. Tel.: 0732 63416; fax: 0732 61340.*

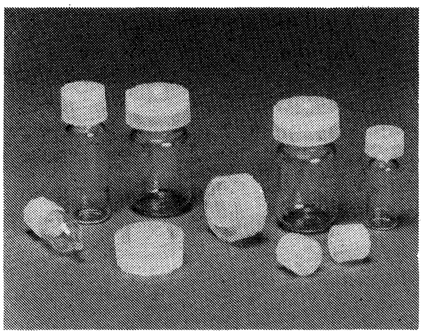

*Chromacol's latest addition to the SNAP-CAP range of disposable polyethylene plugs, the 8-PEPC2. Conventional plastic plugs suffer from two main disadvantages. They can be forced out of the vials by pressure build-up associated with volatile samples, and they can creep out if the inside of the vial neck is wet. The SNAP-CAP plugs have been designed to overcome these problems by having a finned shaft for better sealing, and a special skirt which locks around the vial collar. They are both easy and economical to use. Full details from Chromacol Ltd, Glen Ross House, Summers Row, London N12 0LD.*

### Chromatography at PS Instruments

PS Instruments Ltd is a new company aimed at marketing specialist analytical and preparative scale equipment in the UK. It has concluded an agreement with the Varex Corporation to market the Varex range of chromatography products in this country, including both preparative scale HPLC and GC systems, and the award winning laser light scattering mass detector for HPLC. The company will also be promoting a range of consumable items and accessories for chromatography.

The new company is part of the PS Analytical Group, which is already well known for its range of accessories for elemental analysis, and, most particularly, for its environmental analysers used extensively by the National Rivers Authorities and in the water industry. The PS Instrument Group is based in new premises in Sevenoaks in Kent.

The Chromatography Product Manager of the new company is Ray Dobson who has over 30 years' experience in chromatography, including many years with Philips Scientific in Cambridge. He will be particularly promoting the ELSD detector which offes a solution to many detection problems, for example polymers, carbohydrates, surfactants, steroids, antibiotics, lipids and phospholipids. Being a non-specific detector it can be used in place of an RI detector and it has also found many applications in supercritical fluid chromatography.

*For further details and product information contact Ray Dobson, PS Instruments Ltd, Arthur House, B4 Chaucer Business Park, Watery Lane, Kemsing, Sevenoaks TN15 6QY, UK. Tel.: 63416; fax: 0732 61340.*

### Filter for river water analysers

A system for prefiltering river waters prior to on-line measurements for nitrates and other inorganic components is available from Ionics. The SAMPREP II can be used with most manufacturers' monitors, providing an uninterrupted sample flow. It consists of a proprietary crossflow membrane filter which removes particles down to 0·5 microns, including bacteriological matter.

The filter is easy to clean. The operator simply redirects the sample to a standy-by filter module, while he cleans the one in use.

SAMPREP II has a maximum internal pressure of 80 psi, and can operate on samples in temperatures to 95 °C. A minimum sample flow of 12 to 15 l/min generates a filtrate flow rate from 100 to 300 ml/min.

*Further details from Ionics UK Ltd, Carrington Business Park, Carrington, Urmston, Manchester M31 4DD, UK.*

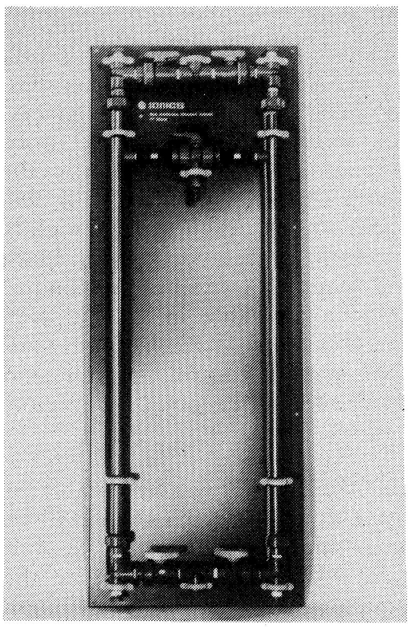

*The SAMPREP II analyser from Ionics UK Ltd. One of these analysers has been installed by Thames Water Authority to filter water prior to analysing for nitrates.*

### Portable mini-lab

A prototype of a new portable labortory is being field tested by Beckman. This assembly, which fits conveniently into a case a little larger than the average briefcase, contains a small, but powerful, centrifuge spinning samples of whole blood at 16 500 rpm and separating plasma from cells within 30 s.

From one drop of blood the portable laboratory equipment can evalaute haemoglobin, haematocrit, glucose, urea, uric acid, cholesterol and triglycerides – a combination which can make this mini-laboratory of value in the evaluation of cardiac risk factors, as well as other applications. Other determinations, such as enzyme studies, could also be achieved within the portable laboratory concept.

It is likely that the new portable mini-laboratory concept will be welcomed in a variety of areas, particularly for veterinary use, for some general and field laboratory applications, domiciliary visits, and in mobile laboratories, private clinics and near patient-testing situations.

*Further information from: Beckman, Progress Road, Sands Industrial Estate, High Wycombe, Buckinghamshire, UK. Tel.: 0494 441181.*

### Differential portable pressure gauges

Two new hand-held pressure gauges are available from Kane-May which provide differential measurement. Each of the two instruments is switchable to give readings over three different scales. The KM5012 differential pressure gauge measures bar,

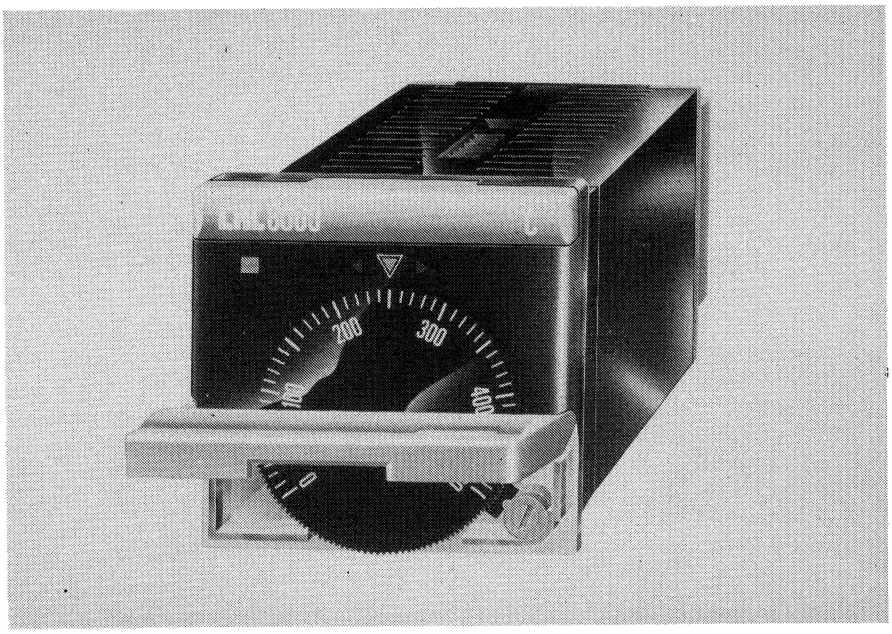

*Space-saving design, with accurate and reliable time proportioning control, are among the features of the 'CAL 8000' series miniature analogue temperature controller, which is believed to be the first miniature analogue instrument to be manufactured using the advanced technology of surface mounted components. Designed to meet a variety of applications, including laboratory packaging, plastics, furnaces, ovens, processing and refrigeration, the unit is 48 mm square (1/16 DIN). Several options are available to maximize the potential of the instrument, including either plug-in or push-on connection and eight temperature ranges between −100°C and 1600°C. Full information from CAL, Bury Mead Road, Hitchin, Hertfordshire SG5 1RT, UK.*

kPa and psig, and the KM5013 measures mbar, inches water gauge, and mm water gauge. Both units are designed for optimum accuracy, linearity and temperature compensation.

Instruments are compact and robust and are supplied complete with tube and coupling in carrying case and are competitively priced.

These instruments are ideal for use in general industrial applications, for preventive maintenance of pipework, to balance and check flow in air conditioning and dehumidifier system, and in processing industries.

*More information from Kane-May Ltd, Swallowfield, Welwyn Garden City, Hertfordshire AL7 1JP, UK. Tel.: 0707 331051; fax: 0707 331202.*

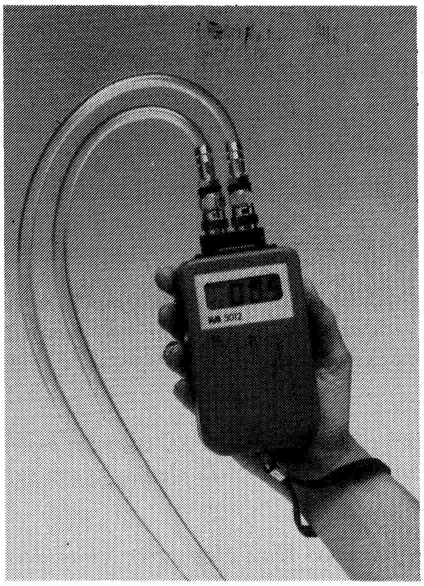

*Differential pressure meters from Kane-May Ltd.*

### Liquid dispensing

Hook & Tucker Instruments has introduced the Compudil D as a fast and accurate means to eliminate routine applications. The Compudil D is a twin syringe dispensing system – essential for the widest possible range of dilution ratios and quicker in use than a one-syringe method. Operation can be via a footswitch or by means of a light, easy to manipulate handset. The latter incorporates a useful miniature LED which illuminates immediately before liquid release.

By turning a four digit thumbwheel switch, any volume can be selected over a recommended working range between 5 and 100% of syringe nominal volume. Moreover, a single digit selector allows nine different operating speeds – with syringe stroke times from 2 to 12 s.

A total of 10 different syringes will fit this instrument. They enable one diluter to be used for dispensing any sample volumes over a 2–5000 μl range, and diluent volumes between 50 μl and 25 ml. Threaded connectors permit quick and easy syringe interchange.

Dispensing accuracy and precision are good – typical performance gives a CV <0·1%. Although it is microprocessor-controlled, there is no need for complicated programming. Once power is on and volume selectors have been set, the instrument is immediately ready for use.

*Further details from Hook and Tucker Instruments Ltd, Vulcan Way, New Addington, Croydon CR0 9UG, UK. Tel.: 0689 43345.*

### Biocompatible LC autosampler

A biocompatible version of the Alcott/Micromeritics Model 728 Autosampler is now available. Designated the 728B, it contains biocompatible materials in key sample transfer areas. The needle block is fashioned from Titanium, connecting tubing is of PEEK, and the user can select an injection valve from a number of different biocompatible materials, such as Titanium, PEEK, or Hastelloy C.

The 728B is based upon the patented, positive displacement design introduced by Micromeritics Instrument Corporation (Norcross, Georgia) in 1976 on one of the first viable autosamplers for HPLC. Automated sample injection is possible without the need for connection to any accessory gas source. The 728B can inject up to 192 times from 64 different samples. All units contain standard RS-232 interface and both fixed and auxiliary contact closures. The autosampler can be used to control an integrator or can be controlled by an integrator, data system or computer. It is designed for interface with any LC system.

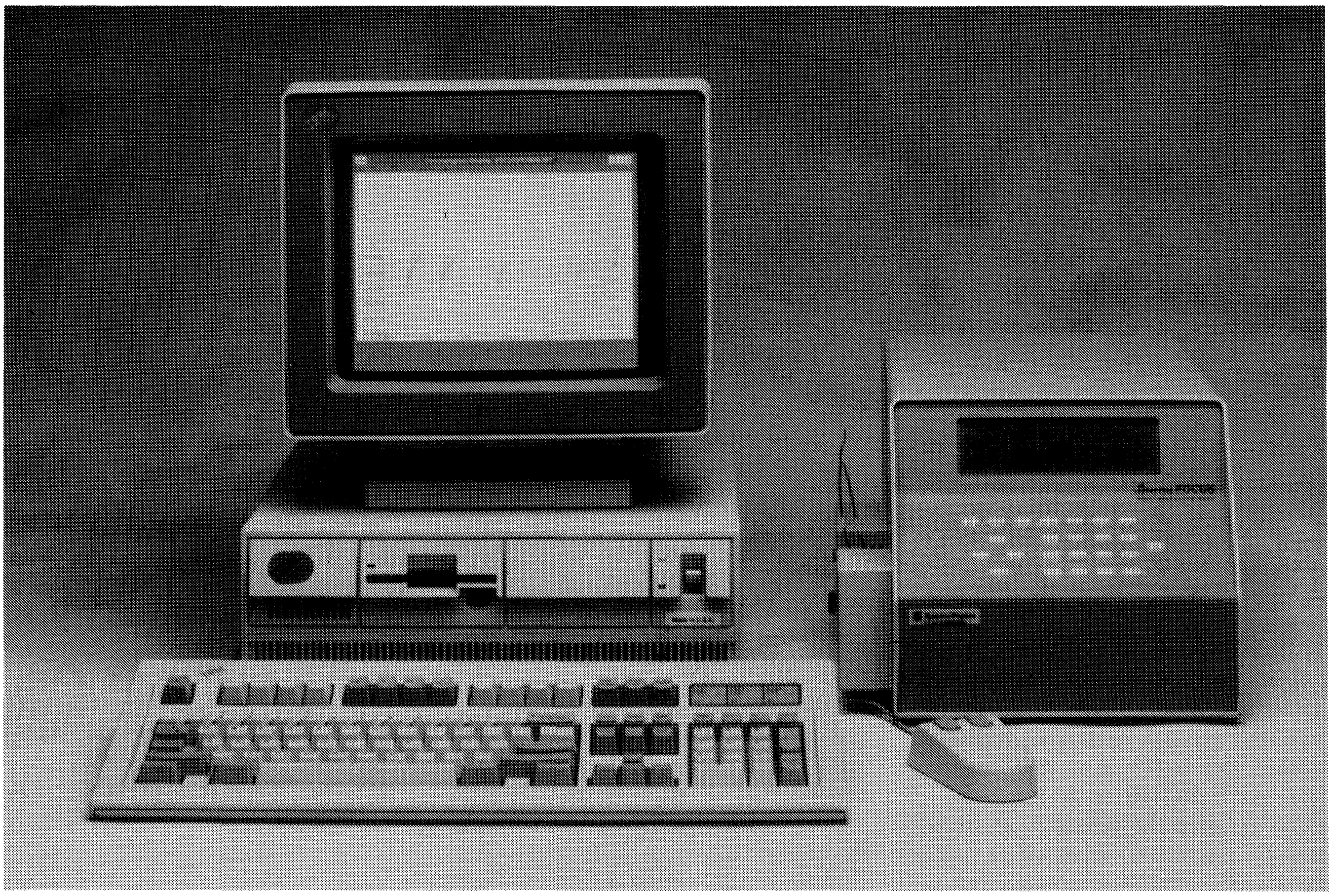

*Spectra FOCUS: a real-time scanning UV-vis detector for liquid chromatography. A 'forward optics' design for high performance offers advantages over current diode array detectors, such as high sensitivity for trace analysis, and versatility for single or multi-wavelength operations. Any LC application can be performed by using an easy-to-change flowcell – from analytical, micro, biotech or prep LC to capillary electrophoresis.*

*Spectra FOCUS Chromatography Software which runs on the IBM PS/2 computer provides multi-tasking operating system features: data acquisition, quantitation/reintegration, LC instrument control, 3-D plotting, peak purity, overlays of spectra, derivatives and multi-wavelength chromatograms.*

*Details from Spectra Physics Autolab Division, Boundary Way, Hemel Hempstead, Hertfordshire HP2 7SH, UK. Tel.: 0442 232322; fax: 0442 68538*

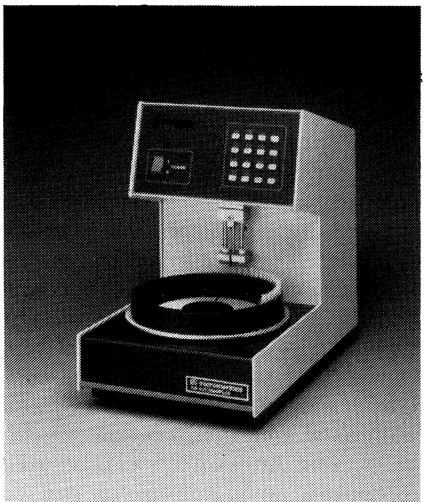

*The biocompatible Alcott/Micromeritics Model 728 autosampler*

*More information from Alcott Chromatography, 5300 Oakbrook Parkway 100, Norcross, Georgia 30093, USA. Tel.: 404 279 2521; fax: 404 279 9153.*

### Accuracy conductivity meter

Compact and lightweight, the Palintest PT115 water conductivity meter is battery operated, has a membrane-switch keypad and uses a new type of composite stainless steel and PVC measurement probe. The meter automatically displays conductivity readings corrected to a standard 25 °C, for all solutions at temperatures between 0 and 50 °C.

The temperature adjustment factor for water, and most aqueous solutions of salts, is 2%, and for most readings the compensation control can be set at this level. However, the effect of temperature on conductivity is not the same for all solutions. For this reason, automatic temperature adjustment can be set between 0 and 2·5%. This will ensure accurate readings with all types of solutions at any temperature. The control setting can be calculated by a simple formula.

The meter has four measurement ranges from 0·1 microsiemens to 199·9 millisiemens. This will cover solutions from distilled water to seawater. Resolution is to 0·1 microsiemens and accuracy is plus or minus 1%. Readings are digitally displayed on a 13 mm high LCD.

Measuring 180 × 83 × 40 mm and weighing 400 g, a single 9 V battery will provide 80 h of continuous use.

*More information from Wilkinson & Simpson Ltd, Palintest House, Kingsway, Team Valley Estate, Gateshead, Tyne & Wear NE11 0NS, UK. Tel.: 091 491 0808; fax: 091 482 5372.*

### Electrodes

Hints and tips on the selection, application, safe use and aftercare of laboratory electrodes are given in a guide available from Radiometer Ltd. This 24-page *Guide to Reliable pH, Ion and Conductivity Measurements* should help electrode users achieve the best possible performance. A comprehensive chart specifies the appropriate pH electrodes for more than 30 given sample types – from 'alkaline solutions', through 'micro samples' and 'surfaces' to 'Yoghurt and curdled milk'.

The *Guide* gives relevant data for all Radiometer Analytical meters, electrodes and accessories for measurement of pH, ion and conductivity. Code references are also included to facilitate ordering.

Although the *Guide* is based on Radiometer's own product ranges, much of the advice is equally appropriate to instruments of all manufacture.

Some hints given – such as the need for proper periodic calibration and maintenance – may always be viewed as 'basic' but are nonetheless often overlooked. These and other tips, such as on the use of ion selective electrodes and on choosing the correct conductivity cell for each application, make the *Guide* a useful laboratory reference.

*Copies are available from Ed Lemon, Manager – Analytical Division, Radiometer Ltd, The Manor, Manor Royal, Crawley, West Sussex RH10 2PY, UK. Tel.: 0293 517599.*

### Paramagnetic oxygen analyser

A continuously operating paramagnetic oxygen analyser for determining the concentration of oxygen in gas streams has been introduced into the US by Applied Automation, a member of the Hartmann & Braun Group. Applications for the Magnos 3 include flue gas monitoring, process monitoring in chemical plants and refineries, and vehicles emissions testing.

New and demanding requirements have been imposed on oxygen-measuring devices to make them applicable in more and more environments and to meet increasingly stringent standards and Magnos 3 offers selectivity, and advanced stability, sensitivity and reliability.

The Magnos 3 is packaged as a complete analyser system with separate electronics and optical compartments – both of which can be purged with air or inert gas. The entire configuration is housed in a dust- and splash-proof fibre-reinforced polyester case.

*Details from Applied Automation (as above).*

### Miniature temperature controllers

Two British miniature temperature controllers have recently been announced. The digital, auto-tuning CAL 9900 series and the analogue CAL 8000 series use surface mount technology, to pack the maximum features into the space-saving 48 $mm^2$ formats. Both units are assembled automatically to ensure reliability at competitively low prices and are designed to combine accuracy with flexibility across a broad variety of industrial processes. With the ability to save up to 75% of the panel space required for earlier types of controllers, they are ideally suited to multi-zone applications needing precise thermal profiles.

The CAL 9900 is a dual setpoint, microprocessor-based unit which automatically auto-tunes its own P, I and D values and permits user selection of any common thermocouple, resistance thermometer, linear voltage or current process transducer input. Users may also make range adjustments within the full temperature span from −200° to +1600 °C and can select display resolution to 1° or 0·1° in units of °C or °F.

The second setpoint can be configured as a PID cool channel with air- or water-cooling options, or alternatively, as a deviation alarm in high, low or out of limits mode, or as an entirely independent process alarm.

The CAL 8000 series is believed to be the first 48 $mm^2$ analogue temperature controller on the market to employ smt. It features accurate and reliable time proportioning control aimed at meeting an extensive range of applications, including packaging, plastics, furnaces, ovens, laboratory and food processing machinery.

It can be calibrated for use with various types of thermocouples or PT100 RTD sensor, and, like the 9900, features an optional second setpoint which can be used in high, low or out of limits mode or in simple heat/cool applications. Other options include plug-in or push-on connection and nine temperature ranges from −100° to +1600 °C.

All units include a five-step LED deviation display and manual reset adjustment. They also offer tamper-proof security features to prevent unauthorised or accidental readjustment. In the case of the 9900, fascia protection to IP54 is provided as standard.

*Details from CAL, Bury Mead Road, Hitchin, Hertfordshire SG5 1RT, UK. Tel.: 0462 436161; fax: 0462 451801.*

